# YAP transcriptionally regulates COX-2 expression and GCCSysm-4 (G-4), a dual YAP/COX-2 inhibitor, overcomes drug resistance in colorectal cancer

**DOI:** 10.1186/s13046-017-0612-3

**Published:** 2017-10-16

**Authors:** Wei Li, Yuanyuan Cao, Jinling Xu, Ying Wang, Weijie Li, Qian Wang, Ziwei Hu, Yaping Hao, Li Hu, Yawen Sun, Guanglin Xu, Guizhen Ao

**Affiliations:** 1 College of life sciences, Nanjing Normal University, Nanjing, China; 2 Jiangsu Key Laboratory of 3D Printing Equipment and Manufacturing, Nanjing Normal University, Nanjing, China; 3 Jiangsu Key Laboratory for Molecular and Medical Biotechnology, College of Life Science, Nanjing Normal University, Nanjing, China; 4 Department of Medicinal Chemistry, School of Pharmacy, Soochow University, Soochow, Jiangsu China

**Keywords:** Yap, COX-2, Colorectal cancer, Resistance

## Abstract

**Background:**

Chemotherapy resistance remains a major challenge in cancer treatment. COX-2 (cyclooxygenase 2) is involved in drug resistance and poor prognosis of many neoplastic diseases or cancers. However, investigations identifying new modulators of COX-2 pathway and searching for new chemicals targeting these valid resistant biomarkers are still greatly needed.

**Methods:**

HCT15, HCT-116, HT-29, COLO205, FHC, IMCE, SW480 cell lines were used to detect the expression of YAP and COX-2. Site-directed mutagenesis, luciferase reporter analysis and ChIP assay were used to test whether YAP activated COX-2 transcription through interaction with TEAD binding sites in the promoter of COX-2. Cell line models exhibiting overexpression or knockdown of some genes were generated using transfection agents. Coimmunoprecipitation was used to detect protein mutual interaction. mRNA and protein levels were measured by qRT-PCR and western blot respectively.

**Results:**

Here, we reported that both YAP and COX-2 were overexpressed in colorectal cancer cells. YAP increased COX-2 expression at the level of transcription requiring intact TEAD binding sites in the COX-2 promoter. YAP conferred drug resistance through COX-2 and its related effectors such as MCL, MDR, Survivin. GCCSysm-4 (G-4), a YAP and COX-2 inhibitor, effectively inhibited both YAP and COX-2 activation, induced apoptosis and decreased viability in Taxol-resistant cells. Inhibition of YAP and COX-2 acted synergistically and more efficiently reduced the resistance of CRC cells than either of them alone.

**Conclusions:**

Our data provide new mechanisms that YAP is a new upstream regulator of COX-2 pathway and plays an important role in conferring resistance in CRC cells. G-4, targeting YAP-COX-2, may be a novel valuable strategy to combat resistance in CRC.

**Electronic supplementary material:**

The online version of this article (10.1186/s13046-017-0612-3) contains supplementary material, which is available to authorized users.

## Background

Adenocarcinoma of the colon and rectum (colorectal cancer, CRC) is the third most common cancer, the fourth most common cause of cancer death, and the second most common cancer in terms of the number of individuals living with cancer five years after diagnosis worldwide. However, the treatment of colorectal cancer is still difficult for therapists. The success of CRC chemotherapy is often compromised by tumor recurrence and acquired resistance. Therefore, broadening our knowledge of molecular signaling including proteins or biomarkers responsible for drug resistance is crucial for developing novel therapeutic strategies to overcome resistance and increase patient survival [1].

Cyclooxygenases (such as COX-1 and COX-2) are prostaglandin (PG) synthases catalyzing the arachidonic acid (AA) to the production of prostaglandins. COX-1 is considered as a housekeeping isoenzyme and constitutively expressed in mammalian cells. In contrast, COX-2 serves as an inducible enzyme and activated by various insults under pathological conditions. The activation of COX-2 results in formation of its major product, PGE_2_, which plays a crucial role in modulating many pathophysiological activities [2, 3]. Recent reports show that COX-2 is overexpressed in many solid tumors, including colorectal cancer, and involved in drug resistance and poor prognosis [4–7].

By using genome microarray and real-time qRT-PCR, many cellular genes have been identified and confirmed as downstream effectors of COX-2. The activation of the COX-2/PGE_2_ pathway can up-regulate the expression of downstream effecters, such as three ABC (ATP-binding-cassette) transporters, MDR1/P-gp (multidrug resistance/P-glycoprotein), MRP1 (multidrug-resistance protein 1), BCRP (breast-cancer-resistance protein), and apoptosis regulating proteins, Survivin, Bcl-2 family and GST system and PKC pathway [3]. In contrast, less upstream regulators of COX-2 pathway are well characterized except NF-kappaB, MAPK, and PI3K/Akt. Therefore, investigations identifying new upstream modulators of COX-2 pathway are urgently needed.

The Hippo signaling pathway has important regulatory effects in organ size and cell proliferation. YAP is one of the two main key downstream effectors of the Hippo signaling pathway and is tightly regulated by some serine-threonine kinases such as mammalian STE20-like protein kinase 1/2 (MST1/2), and large tumor suppressor 1/2 (LATS1/2) [8]. A large body of evidence shows that Hippo pathway plays a critical role in cancer development [9, 10] including CRC and YAP/TAZ has been shown to mediate resistance to chemotherapy in human cancers [11].

In preliminary studies, we found that YAP and COX-2 were up-regulated and positively associated in CRC cells. Considering YAP is a transcriptional coactivator and positive correlation between these two molecules, we hypothesize that YAP activates COX-2 pathway and increases its expression, leading to resistance to drug therapy. In this study, we provide new insights into the idea that YAP enhances COX-2 expression via interactions with transcription factors, TEA domain family members, in the COX-2 promoter. YAP augments a survival pathway through COX-2 up-regulation and initiates therapy resistance to chemotherapeutic agents. YAP and COX-2 act synergistically in keeping drug resistance and anti-apoptosis. Our data demonstrate that YAP is a new upstream regulator of COX-2 pathway and G-4,targeting YAP-COX-2,may be a novel valuable strategy to more effectively combat resistance in CRC.

## Methods

### Cell lines, culture and reagents

HCT15, HCT-116, HT-29, COLO205, FHC, IMCE, SW480 were obtained from the ATCC and cell bank of Shanghai Institute of Cell Biology (Shanghai, China). Cells were cultured in 75- or 150- cm^2^ flasks with Dulbecco’s modified eagle medium (DMEM) supplemented with 10% fetal bovine serum (FBS), 100 U/ml penicillin, and 100 μg/ml streptomycin. Cells were incubated in a 5% CO_2_ incubator at 37 °C.

### Chemicals and reagents

Dulbecco’s modified eagle medium (DMEM) and fetal bovine serum (FBS) (Gibco BRL, USA); trypsin, propidium iodide (PI) and MTT (Sigma Chemical Co., MO, USA); penicillin and streptomycin (Sunshine Biotechnology, Nanjing, China); antibodies to MCL1, MDR, Survivin and horseradish peroxidase (HRP)-linked anti-rabbit IgG were obtained from Cell Signaling (Beverly, MA,USA). Antibodies to COX-2, YAP, LATS1, HRP-linked goat anti-mouse IgG were purchased from Santa Cruz Biotechnology (Santa Cruz, CA, USA). DNA plasmids that encode wild type human YAP (hYAP, CMV2-YAP) and TEAD1 vector (pRK5-Myc-TEAD 1) and YAP, COX-2, LATS1 shRNA were obtained from Addgene (USA). Other agents were the highest quality available in market. G-4 was synthesized in Guizhen Ao’s laboratory.

### Cell viability and apoptosis assay

Cell viability and flow cytometry analysis of cell apoptosis was measured as described previously [2].

### Plasmid construction and site-directed mutagenesis

The DNA of COX-2 [nucleotide (nt) position −800 to −1] promoters were amplified by PCR from genomic DNA extracted from human BxPC-3 cells and subsequently cloned into pGL3-basic luciferase reporter vector (Promega). Site-directed mutagenesis was done using the QuickChange Mutagenesis Kit (Stratagene) according to the manufacture’s protocol.

### Immunoprecipitation and western blot

The immunoprecipitation was done briefly as follows:, the nuclear lysates containing 500 μg protein were incubated with 5 μg primary antibody overnight at 4 °C. Fifty microliters of protein A/G plus-agarose (Santa Cruz Biotechnology) was added and the complex was incubated at 4 °C overnight. The beads were washed three times with high salt buffer (1 M Tris-HCl, pH 7.4, 0.50 M NaCl, and 1% Nonidet P-40) and twice with lysis buffer to eliminate non-specific binding. The immunoprecipitated complexes were released with 2 × sample buffer for Western analysis. Western blot are as described [2].

### Immunofluorescence

This analysis was performed as described previously [12].

### Colony formation assay

This assay was conducted as described previously [12] .

### Promoter analysis

Human COX-2 promoter upstream of the transcription start site 2 kb DNA sequence was obtained from the website of National Institute of Health (http://www.ncbi.nlm.nih.gov). Then binding sites of TEAD in the promoter were found in the COX-2 promoter using the online JASPAR (http://jaspar.genereg.net) software.

### Luciferase reporter analysis

This assay was done as described previously [12]. Briefly, cells cultured in 6-well plates were transfected for 24 h with luciferase reporter plasmid as well as with a Renilla luciferase expression plasmid (Promega) using the Lipofectamine 2000 reagent (Life Technologies). The cells were collected 24 h after transfection, lysed and analysed for luciferase activities using a Dual-luciferase Reporter Assay system (Promega) that already contains an internal control detectable simultaneously with the luciferase reporter gene. The activity of firefly luciferase was normalized by that of the Renilla enzyme.

### Chromatin immunoprecipitation assay

A ChIP-IT Express kit (active motif) was used for chromatin immunoprecipitation (ChIP) analysis of YAP and COX-2 or CTGF/Cyr 61 promoter interaction. In brief, Cells expressing blank vector or YAP were treated with 1% formaldehyde, lysed, and homogenized using a Dounce homogenizer. DNA was shorn by sonication and the sheared chromatin was incubated with 2 mg of IgG (Sigma) or YAP monoclonal antibody (Santa Cruz Biotechnology), followed by PCR by using the primers used for amplification of the COX-2 or CTGF/Cyr 61 promoter. The PCR products were run on a 3% ethidium bromide–agarose gel and visualized under UV.

### Isolation of nuclear and cytosolic fractions

This isolation was operated according to the manufacturer’s instruction using Nuclear and Cytoplasmic Protein Extraction Kit from Byotime Biotechnology Inc.

### Cyclooxygenase enzyme inhibition assay

COX inhibition effect was tested by commercial colorimetric COX inhibitor screening assay kit (Cayman Chem., Co., Cat# 760111). In brief, 150 μl of assay buffer, 10 μl of heme, 10 μl of COX-2 enzyme and 20 μl of G-4 at concentration of 1, 3, 10, 30, and 100 μM were added into a 96-well plate. The plate was shaken carefully and the absorbance of each well was measured at 590 nm using a plate reader (VICTOR X3, PE, MA).

### PGE_2_ measurement

This analysis was conducted as described previously [2].

### Scratch wound healing assay

This assay was done as described previously [12].

### Cell invasion assay

Invasion assay was conducted utilizing transwell chambers following manufacturer’s instructions. 2.5 × 10^5^ cells were plated onto cell culture inserts precoated with matrigel and incubated with 10 μM of G-4 or vehicle for 48 h. The invaded cells were stained with 0.05% crystal violet and counted in five random fields.

### Xenograft mouse model

Animal protocols were approved by the Institutional Animal Care and Use Committee of the Nanjing Normal University, P.R.C.. Four-week-old male nude mice weighing 16 g to 20 g were acquired from Shanghai Silaike Laboratory Animals Co. Ltd., Chinese Academy of Sciences. Cells (5 × 10^6^) were subcutaneously (S.C.) injected into each nude mouse under a sterile environment. After tumor volume reached 100 mm^3^ in size approximately, mice were randomized into five groups as follows: control, Taxol, G-4, celecoxib, Verteporfin treatment group, mice were intraperitoneally administered with above chemicals alone or their combination at doses of 10, 20, 20, 30 mg/kg once every other day.

Tumor size was measured every 7 days using a caliper, and tumor volume was calculated as 0.5 × L × W^2^, with L indicating length and W indicating width. Mice were euthanized at 21 days after injection and specimens from representative tumor tissue were cut with a razor blade and frozen for western blot analysis of YAP and COX-2 expression. The mice were weighed every 3 days and were closely monitored for signs of toxicity.

### Statistical analysis

The values are expressed as the means ± SD from different experiments. The differences in the means between each group were tested by one-way ANOVA followed by Student–Newman–Keuls test (comparisons between multiple groups); *p* < 0.05 was considered statistically significant.

## Results

### YAP and COX-2 were overexpressed in CRC cells and associated with Taxol sensitivity

Both YAP and COX-2 play important roles in cancer development [4–10]. But whether there is a relationship between YAP and COX-2 expression in colorectal cancer cells (CRCs) remains unknown. To address this question, western blot was conducted to detect their expressions in one normal, one immortalized colorectal epithelial cell line and four CRC cell lines.

**Fig. 1:**
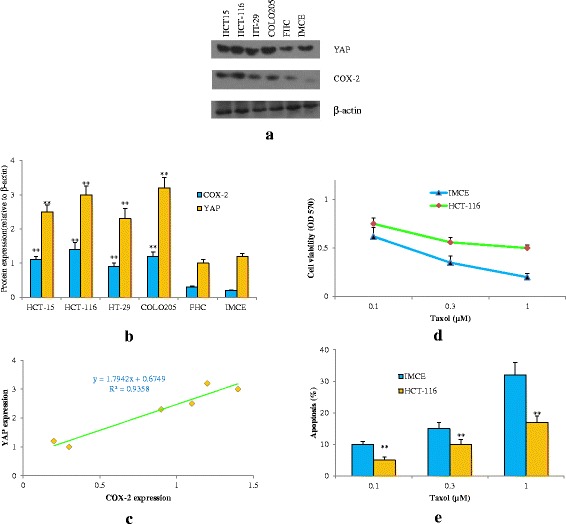
YAP and COX-2 are overexpressed in CRC cells and associated with Taxol sensitivity. **a** YAP and COX-2 expression in HCT15, HCT-116, HT-29, COLO205, FHC and IMCE cells. **b** Summary of YAP and COX-2 expression from three independent experiments. The expression levels of YAP and COX-2 were determined as in A and quantified by densitometry. **P < 0.01 compared with FHC. **c** Correlation analysis between YAP and COX-2 expression. **d** Cell viability of IMCE and HCT-116 cells after treatment with Taxol. Cells were cultured in 96-well plates in the presence or absence of increasing concentrations of Taxol (0.1, 0.3, 1 μM) for 48 h. Cell viability was then determined by using the MTT assay. **e** Apoptosis induced by Taxol in IMCE and HCT-116 cells. Cells were treated with vehicle or Taxol for 48 h, cells (1 × 10^5^) were collected and incubated with Annexin V and PI. The samples were analyzed by flow cytometry. **P < 0.01 compared with IMCE. Data are representative of at least three independent experiments. Error bars represent SD

As shown in Fig. 1a, both YAP and COX-2 levels were low in both normal and immortalized cells (FHC and IMCE), but they were significantly higher in four CRC cell lines examined. We also found that HCT15/HCT-116/HT-29/COLO205 cells, which expressed high levels of YAP protein, also exhibited high levels of COX-2 protein. FHC/IMCE cells expressed low levels of YAP protein concomitant with low levels of COX-2 protein (Fig. 1b). Statistical analysis showed that the coefficient of correlation (R) was 0.966 (Fig. 1c). These data indicated that YAP and COX-2 were up-regulated and highly associated in CRC cells. To determine if both YAP and COX-2 were involved in Taxol sensitivity, we subsequently examined the responses of 2 cell lines IMCE and HCT-116, representing low and high YAP and COX-2 levels, respectively, to increasing concentrations of Taxol, a chemotherapeutic drug commonly used for the treatment of cancers. YAP/COX-2-high HCT-116 had greater cell viability and fewer apoptosis than IMCE cells in response to Taxol (Fig. 1d, e). The above results supported the idea that both YAP and COX-2 were involved in CRC tumor progression as well as chemotherapy sensitivity.

### YAP augmented COX-2 expression in CRC cells

YAP is a major downstream of the Hippo pathway. It functions as a transcriptional co-activator and interacts with TEA Domain (TEAD) DNA binding proteins to initiate the expression of cell-proliferative and anti-apoptotic genes and promote the tumor growth [13].

**Fig. 2:**
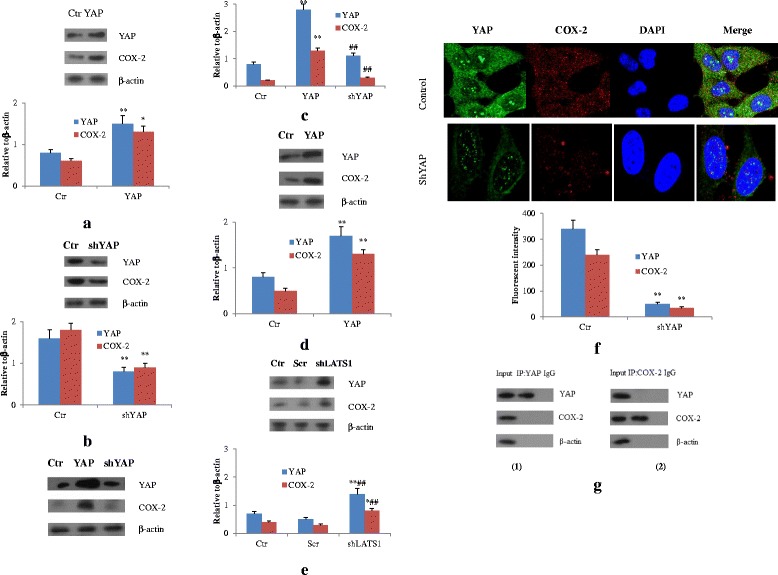
YAP up-regulates COX-2 Expression in both normal and malignant CRC Cells. **a** IMCE cells were transfected with CMV2-YAP plasmid. Immunoblotting using antibodies against YAP, COX-2 were performed. **p < 0.01,*p < 0.05 compared with control. **b** Immunoblotting of YAP, COX-2 was performed in HCT15 cells with knockdown of YAP shRNA. **p < 0.01 compared with control. **c** Immunoblotting of YAP and COX-2 in SW480 cells that were transfected with CMV2-YAP and YAP shRNA successively. **p < 0.01,compared with control; ^##^ *p* < 0.01compared with YAP. **d** YAP and COX-2 was detected by western blot in HEK293T cells transfected with YAP expression vectors. **p < 0.01 compared with control. **e** YAP and COX-2 was detected by western blot in IMCE cells transfected with LATS shRNA. **p < 0.01,*p < 0.05 compared with control;^##^ *p* < 0.01compared with Scramble. **f** Immunofluorescent staining of YAP and COX-2 in HCT15 cells that were transfected with YAP shRNA. **g** Co-IP analysis of interaction of YAP and COX-2. Data are representative of at least three independent experiments. **p < 0.01 compared with control

The above information led to the hypothesis that YAP might activate COX-2 pathway by enhancing COX-2 expression. To confirm this possibility, IMCE cells were transfected with YAP expression plasmid, CMV2-YAP, and the control vector. As expected, elevated YAP in IMCE cells by YAP plasmid up-regulated expression of COX-2 (Fig. 2a). In contrast, shRNA knockdown of YAP in HCT15 cells greatly reduced COX-2 protein levels (Fig. 2b). Moreover, in SW480 cells, COX-2 overexpression induced by CMV2-YAP was attenuated by shRNA knockdown of YAP (Fig. 2c) confirming the direct regulation of COX-2 expression by YAP. Moreover, immunofluorescence revealed that knockdown of YAP by shRNA decreased COX-2 expression in HCT15 cells (Fig. 2f). Similar observations were also found in HT-29 cells (data now shown). Collectively, these studies clearly suggested that YAP increased COX-2 expression in CRC cells.

To examine if COX-2 expression was mediated by YAP in primary cells, human embryonic kidney (HEK293T) cells were used. Results showed that HEK293T cells transfected with CMV-YAP plasmid demonstrated higher COX-2 expression than cells with control vector (Fig. 2d). Additionally, western blots analysis in LATS-disrupted immortal IMCE cells displayed incremental COX-2 expression compared to the control vector-transfected cells (Fig. 2e). Consequently, COX-2 could be up-regulated in several types of cells via expression of a constitutively active form of YAP or by stimulation of endogenous YAP protein that resulted from disruption of Hippo pathway upstream members.

### Interaction between YAP and COX-2

From Fig. 2f, we found that both YAP and COX-2 colocalized in cytoplasma. So we want to know if they have mutual direct interaction. To gain further insight into the relationship between YAP and COX-2 expression, we conducted reciprocal coimmunoprecipitation (Co-IP) experiments and found that YAP could not be readily pulled down by COX-2 and COX-2 could not be pulled down by YAP vice versa (Fig. 2g). This suggested that these two proteins didn’t interact with each other.

### YAP activated COX-2 transcription through interaction with TEAD binding sites in the promoter of COX-2

Having known that there is no interaction between YAP and COX-2 proteins, we next investigated whether YAP could regulate COX-2 expression at the transcriptional level because YAP was a transcriptional co-activator and could initiate the cell proliferation and growth [14].

**Fig. 3:**
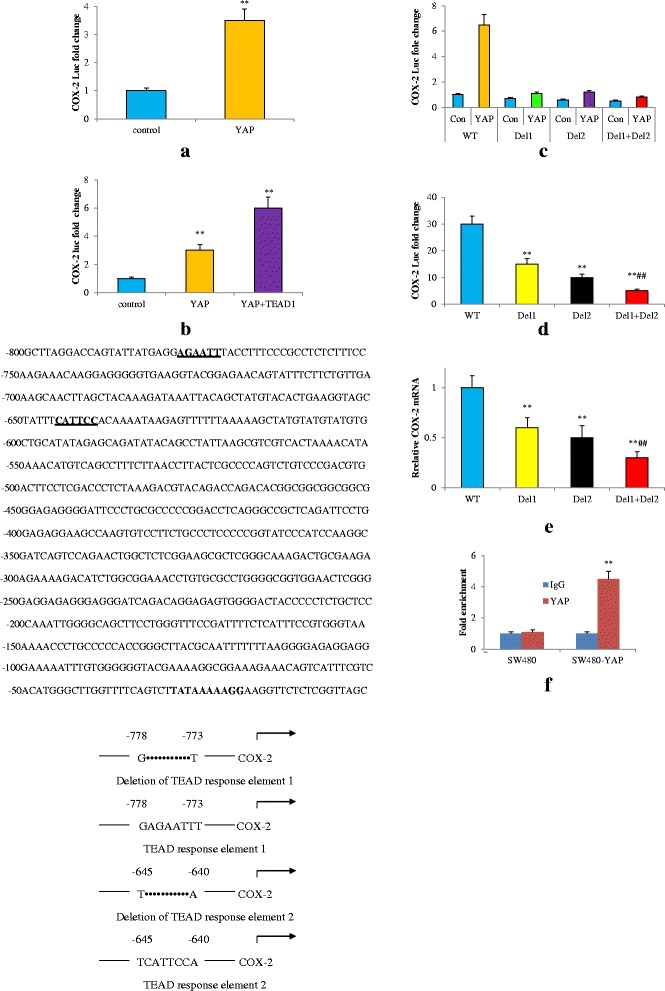
YAP increases COX-2 transcription through intact TEAD response elements. **a** Transfection of COX-2 luciferase promoter reporter into SW480 cells with or without YAP plasmids. COX-2 luciferase reporter activity was measured after 48 h. **b** Co-transfection of COX-2 luciferase promoter reporter with YAP or YAP plus TEAD into 293 T cells; COX-2 luciferase reporter activity was detected after 48 h. **P < 0.01 compared with control cells. **c** Wide type TEAD response elements and deletion of the TEAD response elements were described. Sequence of the COX-2 promoter and two binding sites were identified (bold/underlined, upper panel). Co-transfection of COX-2 luciferase promoters (wide type or deleted in the TEAD binding sites, middle) with YAP or control vector into 293 T cells; COX-2 luciferase reporter activity was detected after 48 h (lower panel). **d** COX-2 luciferase reporter activity (wide type or deleted in the TEAD response elements) was detected after 48 h in HCT-116 cells. **e** mRNA levels of COX-2 were determined by qRT-PCR in HCT-116 cells of d. **P < 0.01 compared with WT, ^##^ *P* < 0.01 compared with Del1 or Del2. **f** ChIP analysis of YAP interaction with the COX-2 promoter in vivo. **P < 0.01 compared with SW480. Data are representative of at least three independent experiments. Error bars represent SD

Analysis of the human COX-2 proximal promoter region reveals two TEAD response elements located around −778 to −773 (AGAATT) and −645 to −640 (CATTCC) of base pairs upstream of the transcription start site. The COX-2 promoter including these TEAD response elements from the transcription start site was cloned and fused to a luciferase cDNA and cloned to the pGL3 vector and then were transfected into SW480 cells. When the YAP plasmids were introduced into SW480 cells, we found that YAP activated the luciferase activity of COX-2 promoters three and a half fold (Fig. 3a).

As YAP has been shown to bind to TEAD transcription domains, we wanted to confirm whether YAP and TEADs could transactivate COX-2 promoter-luciferase construct in non-tumor cells. Therefore, the COX-2 promoter was co-transfected with either YAP alone or with both YAP/TEAD1 into 293 T cells, COX-2 Luciferase activities were increased more than three fold by YAP, while cotransfection of YAP with TEAD1 significantly enhanced the COX-2 transcriptional activity by nearly six fold (Fig. 3b). This indicated that YAP and TEAD were both required to induce COX-2 transcription. To examine if the TEAD response elements in the COX-2 promoter were essential for the arousal of COX-2 by YAP, the deletion of the TEAD response elements was generated in the COX-2 promoter using site-directed mutagenesis as shown in Fig. 3c. Our luciferase assay showed that deletion of the TEAD response elements in the COX-2 promoter greatly diminished COX-2 transcriptional activity in 293 T cells. Similarly, in HCT-116 cells, COX-2 transcriptional activity was significantly reduced, when deletion of the TEAD response elements in the COX-2 promoter was carried out. Accordingly, there was a reduced COX-2 mRNA level after deletion, which was in concert with decreased COX-2 transcriptional activity (Fig. 3d, e). Finally, our ChIP assay showed that YAP could be coimmunoprecipitated with the COX-2 promoter DNA in vivo (Fig. 3f). These data indicated that the interaction of YAP with TEAD response elements was critical for its activation of COX-2 transcription.

### YAP activated COX-2 pathway and regulated cell survival and colony formation abilities

**Fig. 4:**
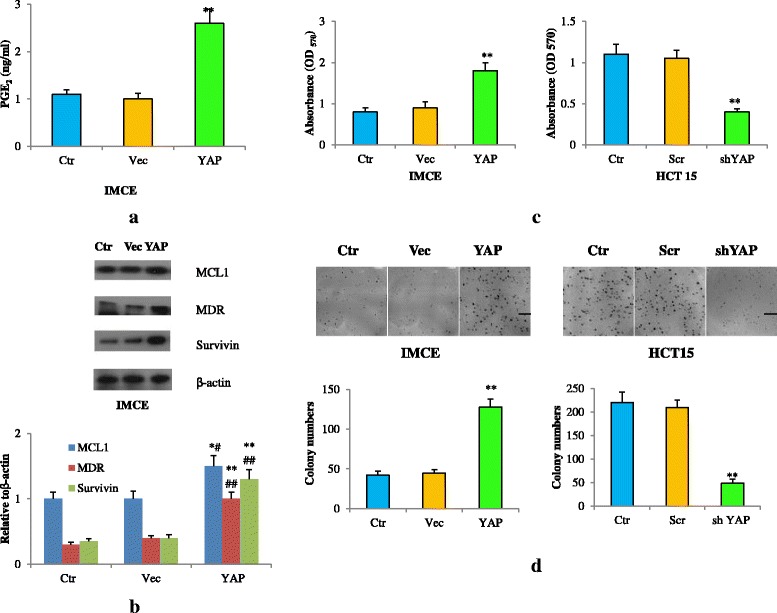
YAP activates COX-2 signaling and mediates cell survival and colony forming abilities. **a** PGE_2_ levels in IMCE cells transfected with or without YAP plasmid. **P < 0.01 compared with control cells. **b** MDR, Survivin, MCL1 protein levels determined by western blot in IMCE cells transfected with or without YAP plasmid. **p < 0.01,*p < 0.05 compared with control;^##^ *p* < 0.01,^#^ *p* < 0.05 compared with empty Vector. **c** Cell viability of IMCE cells transfected with or without YAP plasmid (left panel). Cell viability of HCT15 cells transfected with or without YAP shRNA (right panel). **P < 0.01 compared with control cells. **d** Representative images of spheres in IMCE cells with or without YAP induction (top left); Representative bar graph demonstrating the sphere numbers in IMCE cells with or without YAP induction (low left). Representative images of spheres in HCT15 cells with control and YAP knockdown (YAP shRNA) (top right); Representative bar graph demonstrating the sphere numbers in HCT15 cells with control and YAP knockdown (YAP shRNA) (low right). Scale bar: 5 mm. **p < 0.01 compared with control. All data are representative of at least three independent experiments. Error bars represent SD

It has been shown that YAP is able to cause an increase of COX-2 expression. We next wanted to examine if YAP also increased its downstream effecters. Increased expression of YAP in IMCE cells significantly induced its catalyzed product PGE_2_ increase in concert with the increase in anti-apoptotic protein MCL1. Further, YAP increased and sustained MDR and Survivin expression (Fig. 4a, b). To determine the biological functions of YAP induction in CRC cells, we conducted several assays and found that elevated YAP expression in IMCE cells increased its proliferation (Fig. 4c), and colony forming ability (Fig. 4d). In contrast, down-regulation of YAP by shRNA in HCT15 cells decreased cell viability (Fig. 4c) and greatly reduced clonogenic ability (Fig. 4d). These suggested that YAP was required for tumor cell survival and maintenance which probably embraced activation of COX-2 signaling.

### YAP conferred therapy resistance in CRC cells through COX-2 activation

**Fig. 5:**
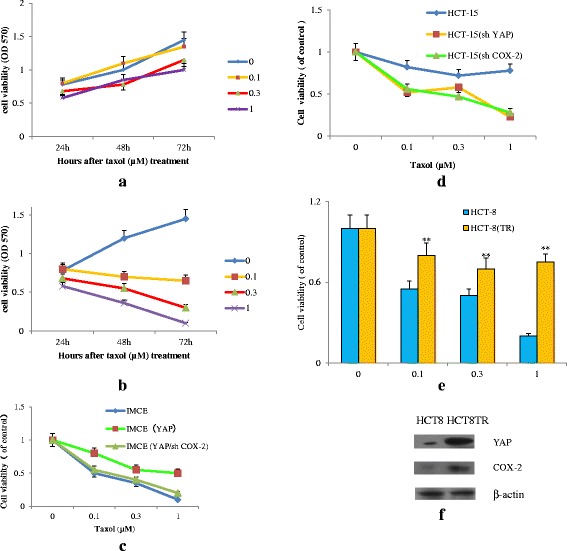
YAP mediates chemoresistance in CRC cells. **a** Cell viability of HCT15 cells treated with indicated concentrations of Taxol for 24, 48, 72 h. **b** Cell viability of IMCE cells treated with indicated concentrations of Taxol for 24, 48, 72 h. **c** Cell viability of IMCE cells and IMCE cells with YAP transfection and treated with indicated concentrations of Taxol for 72 h. **d** Cell viability of HCT15 cells, HCT15 cells with YAP shRNA or COX-2 shRNA transfection and treated with indicated concentrations of Taxol for 72 h. **e** Cell viability in HCT8 and Taxol-resistant HCT8 (HCT8 TR) cells under the treatment of Taxol at different concentrations. **p < 0.01 compared with HCT8 cells. **f** Immunoblotting for YAP and COX-2 was performed in HCT8 and Taxol-resistant HCT8 (HCT8 TR) cells. Data are representative of at least three independent experiments. Error bars represent SD

Expressions of YAP and COX-2 have been both reported to increase in tumor cells and participate in drug sensitivity as shown in Fig. 1. We next want to know whether YAP-regulated COX-2 was essential for chemotherapy response in CRC cells. HCT15 and IMCE had high or low levels of YAP/COX-2 expression. As shown in Fig. 5a, b, HCT15 cells with high YAP/COX-2 expression had a poorer response to Taxol than IMCE cells with low YAP and COX-2.

To further examine the direct relationship between YAP/COX-2 and chemotherapy response, IMCE cells with higher YAP/COX-2 expression revealed worse response on exposure to Taxol than parental cells (Fig. 5c). In addition, down-regulation of YAP in HCT15 cells drastically enhanced cell sensitivity to Taxol than its parental cells (Fig. 5d). What’s more, in the stable Taxol-chemoresistant CRC cells HCT8 (HCT8 TR), there were high expressions of YAP and COX-2 compared to their parental cells, which was consistent with their significant resistance to Taxol treatment (Fig. 5e, f). Furthermore, as shown in Fig. 5c, when we knocked down COX-2 in IMCE (YAP) cells (i.e., IMCE(YAP/shCOX-2)), they became more sensitive to Taxol treatment than intact IMCE (YAP) cells. These results indicated that YAP- induced COX-2 expression augmented chemoresistance in CRC cells.

### G-4 induced phosphorylation and cytosol localization of YAP

The Hippo cascade promotes phosphorylation and cytosolic retention of the YAP, leading to degradation of YAP, while dephosphorylation of YAP drives YAP to enter the nucleus and interact with TEA Domain (TEAD) DNA binding proteins to promote cell growth [15].

**Fig. 6:**
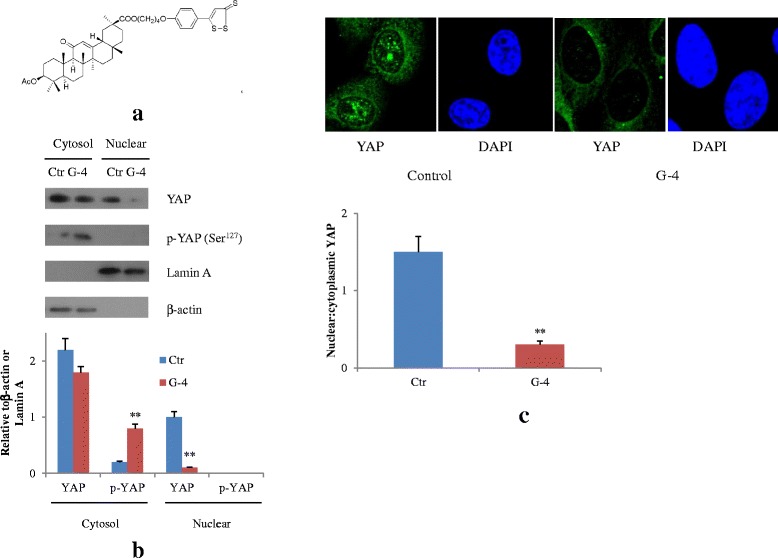
G-4 inactivates YAP in colorectal cancer cells. **a** Structure of G-4. **b** G-4 treatment (6 h, 10 μM) induces YAP phosphorylation in cytosol and decreases YAP levels in nucleus of HCT15/Tax cells. **p < 0.01 compared with control. **c** G-4 (6 h, 10 μM) decreases YAP nuclear localization in HCT15 /Tax cells. YAP subcellular localization was determined by immunofluorescence staining for endogenous YAP (green) along with DAPI for DNA (blue). Data are representative of at least three independent experiments. **p < 0.01 compared with control

G-4 is a newly developed hydrogen sulfide-releasing drug in our lab with remarkable inhibition on COX-2 (see structure in Fig. 6a). Recently, hydrogen sulfide-releasing drugs have been shown to exhibit inhibitory effect on the growth of human cancer cells. Considering the above relationship between Hippo-YAP and COX-2 pathway in cancer progress, we wanted to know whether G-4 had effect of on these two pathways. First, we tested whether G-4 affected the phosphorylation status of YAP in nuclear and cytosol fractions of HCT8 and HCT15 taxol-resistant cells. We found an increase in the level of p-YAP in cytosolic fractions and a decrease of YAP in nucleus after G-4 treatment (Fig. 6b, Additional file 1: Figure S1A). The same phenomenon was observed under immunofluorescence as shown in Fig. 6c and Additional file 1: Figure S1B. These data demonstrated that G-4 could potently inactivate YAP by inducing phosphorylation and cytosol localization.

### G-4 impaired YAP-TEAD complex in CRC cells

**Fig. 7:**
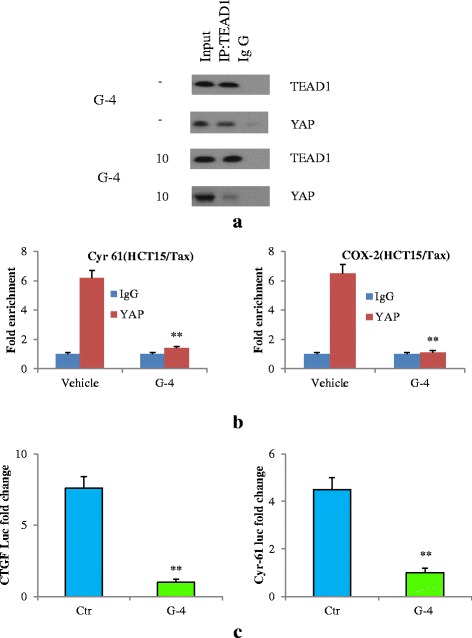
G-4 (10 μM) disturbed YAP-TEAD interaction. **a** G-4 treatment disturbed the YAP-TEAD1 interaction in the nucleus of HCT15/Tax cells. The YAP-TEAD1 interaction was probed in cells 4 h after G-4 treatment and in untreated cells using co-IP. **b** ChIP analysis of YAP interaction with the Cyr 61 and COX-2 promoter in HCT15/Tax cells. YAP was examined in cells 4 h after G-4 treatment and in untreated cells. **P < 0.01 compared with vehicle group. **c** Cyr 61 and CTGF luciferase reporter activity was measured after treatment with G-4 for 48 h. The fold changes in luciferase activity were calculated by normalizing untreated cells with G-4-treated cells. Data are representative of at least three independent experiments. Error bars represent SD. **P < 0.01 compared with control cells

After confirming the effect of G-4 on YAP localization, we next examined the interaction between YAP and TEAD. Thus, we first verified the existence of a YAP-TEAD interaction in HCT8 and 15/Tax cells and tested the effect of G-4 treatment on them. To examine the existence of a YAP-TEAD1 interaction, we adopted coimmunoprecipitation (co-IP) assays. In this assay, TEAD1 was precipitated from the nuclear fraction by an anti-TEAD1 antibody and the presence of YAP in the precipitate was monitored using anti-YAP antibody in the final immunoblot. With the aid of this technique, we discovered a YAP-TEAD interaction in the nuclear fraction of HCT8 and 15/Tax cells (Fig. 7a, Additional file 2: Figure S2A) and next found that treatment with G-4 conspicuously reduced the amount of YAP recovered in the immunoprecipitates of TEAD1, indicating that G-4 clearly reduced the YAP–TEAD interaction. Therefore, we bore out the interaction between YAP and TEAD1 in HCT8 and 15/Tax cells and identified G-4 as an inhibitor targeting the physical interactions between YAP and TEAD. To further understand the role of YAP/TEAD transcriptional activity in the effect of G-4 on HCT8 and 15/Tax cells, we compared the level of YAP DNA binding activity in control cells with that of G-4-treated cells. Consistent with previous observation of reduced interaction between YAP and TEAD, our ChIP assay showed a significant decrease in the amount of YAP that was coimmunoprecipitated with the Cyr61 promoter DNA in G-4-treated cells compared to the untreated cells (Fig. 7b, Additional file 2: Figure S2B). Furthermore, we found that YAP significantly activated the luciferase activity of both Cyr61and CTGF promoters (Cyr61-luc or CTGF-luc) in control cells, whereas treatment with G-4 abolished YAP’s activation of the Cyr61 and CTGF promoters (Fig. 7c).

### G-4 attenuated COX-2 expression and inactivated enzyme catalyzation

**Fig. 8:**
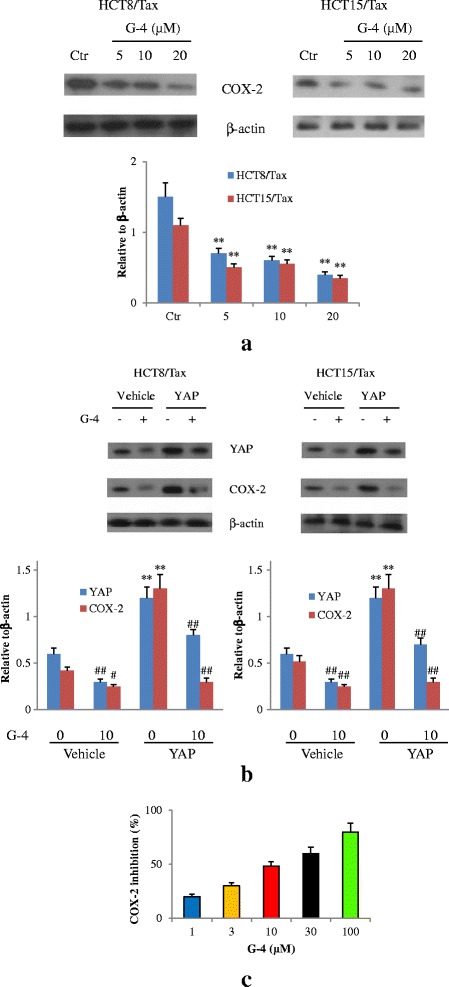
G-4 inhibited COX-2 expression and enzyme activity. **a** COX-2 expression in HCT8 and 15/Tax cells treated with different concentrations of G-4 (5–20 μM) for 24 h. **P < 0.01 compared with control cells. **b** YAP and COX-2 expression in HCT8 and 15/Tax cells transfected with YAP expressing plasmids following G-4 treatment (10 μM). **P < 0.01 compared with vehicle group. ^#^ *P* < 0.05, ^##^ *P* < 0.01 compared with G-4 0 μM group. **c** COX-2 enzyme activity inhibition by different concentrations of G-4 at 1, 3, 10, 30, and 100 μM. Data are representative of at least three independent experiments. Error bars represent SD

COX-2 plays an important role in both inflammatory and cancer process. Because G-4 has been shown to exhibit significant anti-inflammatory properties with inhibition of COX-2 [16], we want to know whether it has effect on COX-2 expression in cancer cells. As shown in Fig. 8a, COX-2 expressed at a high level in untreated HCT8 and 15/Tax cells. Upon G-4 treatment, significant reduction of COX-2 expression was observed compared to the G-4-untreated cells in a concentration-dependent manner. Considering the YAP inhibition of G-4 might result in low COX-2 expression, we use YAP-expressing vectors to reverse the low level of YAP. FigURE 8b showed that after transfection, YAP level was increased to a high level comparable to the pretreatment situation, while the low COX-2 expression did not change significantly following YAP increase. These data suggested that G-4 affected COX-2 expression independent of its YAP inhibitory activity. Further COX-2 enzyme inhibition assay was carried out to determine whether it had direct inhibitory effect on COX-2 enzyme. As shown in Fig. 8c, G-4 inhibited COX-2 enzymes in a dose-dependent manner. Collectively, G-4 affected not only COX-2 expression at posttranscriptional level but also COX-2 enzyme catalytic activity. Together with the above-mentioned YAP inactivation, G-4 can be identified as a dual inhibitor of YAP and COX-2.

### G-4, a dual inhibitor of YAP and COX-2, increased apoptosis and inhibited migration/invasion

**Fig. 9:**
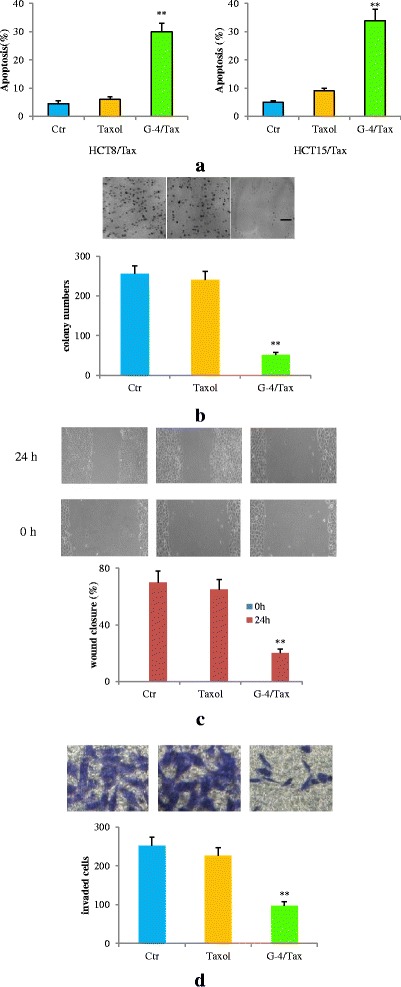
G-4 (10 μM) induced apoptosis and suppressed cell colony formation, migration and invasion. **a** Flow cytometric analysis for apoptosis. HCT8/Tax and HCT15/Tax cells were treated with G-4 for 48 h. Taxol-treated cells were used in parallel as a control. **b** Effect of G-4 on cell colony formation. HCT15/Tax cells were seeded into 6-well plates and 9 days later, the colonies were stained with crystal violet, photographed (upper panel) and counted (lower panel). Scale bar: 5 mm. **c** Effect of G-4 on cell migration in HCT15/Tax cells. Cells were seeded into 6-well plates at 70–80% confluence. Cell migration was monitored by optical inspection for 24 h using a microscope and pictures were taken at 0 and 24 h (upper panel) and quantified (lower panel). **d** Effect of G-4 on cell invasion in HCT15/Tax cells. Invasion assay was conducted utilizing transwell chambers. The invaded cells were photographed (upper panel) and quantified (lower panel). Data are representative of at least three independent experiments. Error bars represent SD. **P < 0.01 compared with Taxol. G-4 and Taxol was applied at10 and 1 μM respectively. HCT8/Tax and HCT15/Tax:Taxol- resistant HCT8 and HCT15 cell lines. G-4: GCCSysm-4, Tax:Taxol

Now that G-4 exhibited characteristics of dual inhibition on YAP and COX-2, we next sought to test its impact on resistant HCT8 and 15/Tax cells. As shown in Fig. 9a, Taxol caused apoptosis in 6% of HCT8/Tax cells and 9% of HCT15/Tax cells. G-4/Taxol increased the apoptosis percentage from 6% to 30% in HCT8/Tax and from 9% to 34% in HCT15/Tax cell cultures respectively. We also found that their combination had similar effects on decreasing cell viability, more efficacious than Taxol in HCT8 and 15/Tax cells (Additional file 3: Figure S3A). Coincidently, combination of G-4 and Taxol hampered the clonogenic formation, resulting in a remarkable decline in the number of colonies (Fig. 9b, Additional file 3: Figure S3B). In cell migration and invasion assays, results showed that G-4/Taxol markedly inhibited the wound closure (15%) in comparison with that of Taxol group (65%) for 24 h in HCT15/Tax cells (Fig. 9c). Similar results were observed using HCT8/Tax cells (Additional file 3: Figure S3C). The results from chamber invasion assay demonstrated that HCT15/Tax cell invasion was significantly decreased by ~50% upon combination treatment compared to Taxol alone (Fig. 9d). The similar results were obtained using HCT8/Tax cells (Additional file 3: Figure S3D). These results suggest that G-4 is capable of inhibiting cell growth and mobile abilities of HCT8 and 15/Tax cells and overcoming its resistance against Taxol.

### G-4 changed downstream effectors of YAP and COX-2

**Fig. 10:**
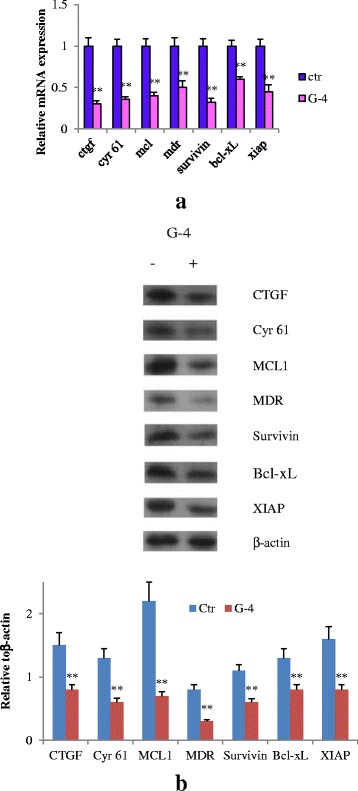
G-4 (10 μM) changed the expression of downstream effectors. **a** Cells were treated with G-4 for 24 h and expressions of CTGF, Cyr61, MCL, MDR, Survivin, Bcl-xL, XIAP were determined by quantitative RT-PCR and were normalized to control. **b** Protein levels of CTGF, Cyr61, MCL, MDR, Survivin, Bcl-xL, XIAP were confirmed by Western blot. Data are representative of at least three independent experiments. Error bars represent SD. **P < 0.01 compared with control

To investigate the underlying molecular mechanism of the anti-resistance properties of G-4, we examined the expression of downstream effectors utilizing realtime PCR and western blot. Figure 10a, b revealed that the mRNA and protein levels of CTGF, Cyr61, MCL, MDR, Survivin, Bcl-xL, XIAP were obviously decreased by G-4.

### YAP and COX-2 were required for the effect of G-4 and acted synergistically to overcome the resistance of HCT8 and 15/tax cells

**Fig. 11:**
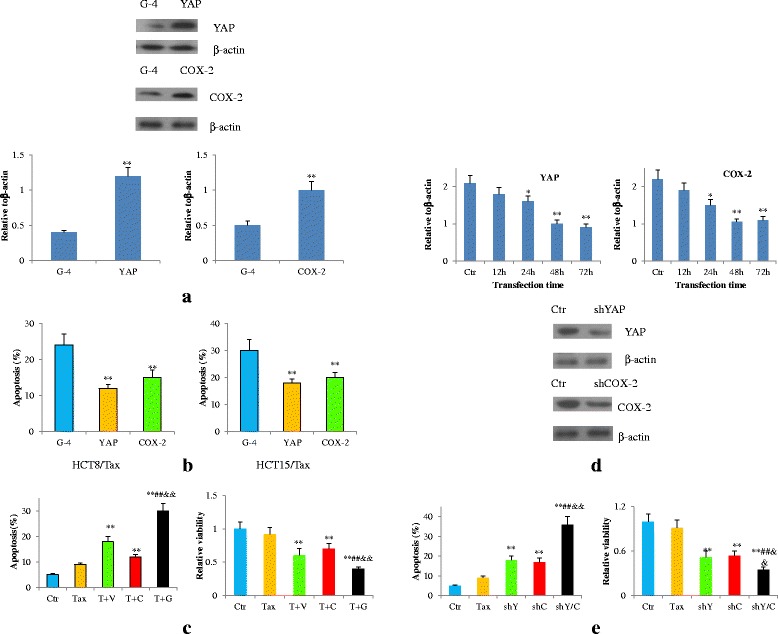
YAP and COX-2 were essential for the effect of G-4 (10 μM) and acted synergistically to overcome the resistance. **a** Western blot analysis of YAP and COX-2 in YAP or COX-2 expressing vector-transfected HCT15/Tax cells treated with G-4 (10 μM) for 48 h. **P < 0.01 compared with G-4. **b** Flow cytometric analysis for apoptosis in YAP or COX-2 expressing vector-transfected cells treated with G-4 (10 μM) for 48 h. Left panel: HCT8/Tax cells; right panel: HCT15/Tax cells. **P < 0.01 compared with G-4. **c** Apoptosis and cell viability of HCT15/Tax cells treated with Taxol, Taxol plus Verteporfin (T + V), Taxol plus Celecoxib (T + C), Taxol plus G-4 (T + G) respectively for 48 h. **P < 0.01 compared with Taxol, ^##^ *P* < 0.01 compared with T + V, ^&&^ *P* < 0.01 compared with T + C. (Taxol: 1 μM; Verteporfin, Celecoxib, G-4 are all 10 μM). **d** YAP or COX-2 expression after shRNA transfection for different time (upper panel), and western blot of YAP and COX-2 (lower panel) after 48 h shYAP or shCOX-2 transfection in HCT15/Tax cells. *P < 0.05, **P < 0.01 compared with control. **e** Apoptosis and cell viability of HCT15/Tax cells after shYAP or shCOX-2 was introduced. **P < 0.01 compared with Taxol, ^##^ *P* < 0.01 compared with shYAP, ^&&^ *P* < 0.01 compared with shCOX-2. All data are representative of at least three independent experiments. Error bars represent SD. Tax: Taxol, VP: verteporfin, Cel: celecoxib, G-4: GCCSysm-4.

To determine whether the YAP and COX-2 were the critical determinants of drug-induced resistance in these cells, we assessed the effects of YAP and COX-2 expressing vectors on apoptosis induced by G-4. The YAP or COX-2 vector effectively up-regulated expression of the target proteins, compared with the control vector in G-4-treated HCT8 and 15/Tax cells (Fig. 11a). Then, these cells were exposed to G-4 again and were further analyzed by flow cytometry and MTT assays. G-4 induced apoptosis in 24% of intact HCT8/Tax cells and 30% of HCT15/Tax cells. Transfection with YAP vector decreased the apoptosis percentage from 24% to 12% in HCT8/Tax cells and 30% to 18% in HCT15/Tax cells. The COX-2 vector had similar effects and decreased apoptosis in HCT8 and 15/Tax cells too (Fig. 11b). We also found that both vectors had similar effects on increasing cell viability in HCT8 and 15/Tax cells (Additional file 4: Figure S4A). G-4 induced apoptosis more significantly than either VP (a YAP inhibitor) or Cel (a selective COX-2 inhibitor) alone in HCT8 and HCT15/Tax cells. Simultaneously, G-4 resulted in more decreases of viability of HCT8 and HCT15/Tax cells than VP or Cel alone (Fig. 11c, Additional file 4: Figure S4B). According to these results, both YAP and COX-2 are important regulators in G-4-reduced drug resistance in HCT8 and 15/Tax cells.

To further determine whether the down-regulation of YAP and COX-2 were synergistic in apoptosis of these cells, we assessed the effects of shRNA targeting YAP and COX-2 on apoptosis sensitivity in HCT8 and 15/Tax cells. The shRNA targeting YAP or COX-2 effectively down-regulated expression of the target proteins, compared with the control shRNA (Fig. 11d). Then, the HCT8 cells were exposed to Taxol after shRNA treatment and were further analyzed by flow cytometry and MTT assays. Transfection with shRNA targeting YAP increased the apoptosis percentage from 6% to 15% in HCT8/Tax cells and 9% to 18% in HCT15/Tax cell cultures (Fig. 11e, Additional file 4: Figure S4C). The shRNA targeting COX-2 had similar effects and increased apoptosis in HCT8 and 15/Tax cells too. The cotransfection of these two shRNAs synergistically increased apoptosis in HCT8 and 15/Tax cells. We also found that both shRNAs had synergistic effects on decreasing cell viability in HCT8 and 15/Tax cells (Fig. 11e, Additional file 4: Figure S4C). Based on these results, YAP and COX-2 are important synergistic regulators of drug resistance in HCT8 and 15/Tax cells, which is consistent with G-4’s more potency on them than a YAP and COX-2 inhibitor alone.

### G-4 inhibited tumor growth in xenograft mouse model

**Fig. 12:**
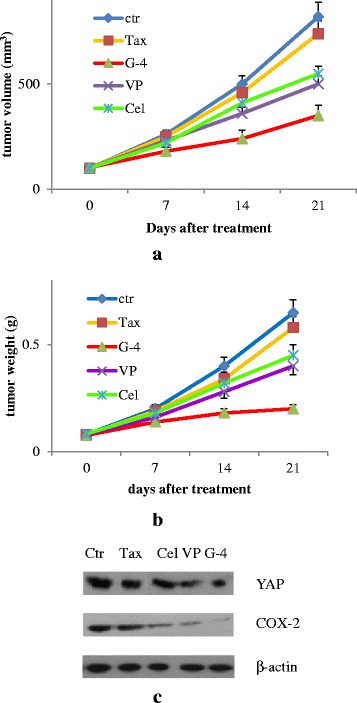
G-4 inhibited in vivo tumor growth. Mice were randomly divided into five groups: control, Taxol, G-4/Tax, verteporfin/Tax and celecoxib/Tax. HCT15/Tax cells (5 × 10^6^) were subcutaneously (SC) injected into nude mice. Mice were intraperitoneally administered with Taxol, G-4, VP, celecoxib at a dose of 10, 20, 30, 20 mg/kg alone or their combination. Tumor size was measured every 7 days using a caliper, and tumor volume was calculated as 0.5 × L × W^2^, with L indicating length and W indicating width. G-4 significantly decreased in vivo tumor growth according to the results of calculated tumor volume **a** and tumor weight **b**. **c** YAP and COX-2 expression in G-4-treated nude mice samples. (*n* = 5 each group) **p < 0.01 compared with Taxol group, ^##^ *p* < 0.01, ^&&^ *p* < 0.01 compared with VP/Tax and Cel/Tax group respectively. Tax: Taxol, VP: verteporfin, Cel: celecoxib, G-4: GCCSysm-4

Male athymic nude mice, allowing for the development of subcutaneous tumors, were used to demonstrate whether G-4 was able to reduce the tumor volume/weight in mice where the tumor was already detectable. They were observed closely, there were no overt signs of toxicity, based on the average weights of the mice in each group at the beginning and end of the study and overall appearance of the treated animals. The G-treated mice showed a more considerable reduction in tumor volume and weight than other treated mice (Fig. 12a, b). Compared with the control group, VP, celecoxib and G-4 reduced the tumor weight with percentage of 39%, 31% and 69% respectively, suggesting that G-4 had an effect on tumor proliferation in mice where the tumor was detectable at the time of treatment. In addition, the levels of YAP, COX-2 in mice tumors were significantly reduced by the therapy of G-4 (Fig. 12c). Hence, G-4 could counteract the obtained chemo-resistance in CRC tumors via suppression of both COX-2 and YAP in vivo.

## Discussion

Although tremendous progress has been made toward understanding the molecular mechanism underlying colorectal cancer development and treatment, the drug-resistance is still an unconquerable barrier for successful survival. Therefore, the identification of pathway networks including proteins or biomarkers responsible for drug resistance is still crucial for successful colorectal cancer treatment [17].

In this study, we described for the first time that YAP up-regulated COX-2 expression at the level of transcription through intact TEAD binding sites. Both YAP and COX-2 were overexpressed in CRC cells and associated with acquired chemo-resistance in cell lines. YAP increased COX-2 expression and exacerbated the effect of therapy resistance. VP, a novel YAP inhibitor, effectively inhibited both YAP and COX-2 expression and sensitized CRC cells to Taxol treatment. Additionally, combination of VP and COX-2 inhibitor celecoxib more efficiently reduced the viability of CRC cells than either of them alone. Our data point to the idea that the YAP-TEAD-COX-2/PGE_2_ signaling pathway is an important regulator of the Taxol response in CRC cells, as well as that dual YAP and COX-2 inhibition is a novel therapeutic approach to treat drug-resistant colorectal cancers.

COX-2 (cyclooxygenase 2), an inducible form of the enzyme that catalyses AA (arachidonic acid) into prostaglandins (PGs), is associated with inflammatory diseases and carcinogenesis. Recently, COX-2 is also found to be involved in drug resistance and poor prognosis of many neoplastic diseases or cancers [18–20].

Although COX-2 has been shown to be associated with drug resistance, the upstream regulators mediating COX-2 resistance remain to be further identified. Till now, few molecules have been identified and confirmed as upstream modulators of COX-2. They are NF-kappaB, MAPK and PI3K/Akt [21, 22]. However, in this study, we have characterized and confirmed YAP as an upstream regulator of COX-2. First, we had shown that overexpression of YAP in IMCE cells caused increased protein levels of COX-2 and release of its catalyzed product PGE_2_. Second, we had also demonstrated that knockdown of YAP in YAP-overexpressing HCT15 reduced the increased COX-2 protein levels. Third, we had shown that YAP activated COX-2 at the level of transcription through a TEAD binding site in the COX-2 promoter.

Recently, COX-2 is also found to mediate drug resistance through regulation of *survivin, bcl-2* family, *mdr1* genes [23–28]. In this study, we found that YAP overexpression resulted in up-regulation of downstream effecters of COX-2, MDR, MCL1, Survivin, all of which participated in the progression of drug resistance. These up-regulations are compatible with previous observations that COX-2 is associated with a poor prognosis in cancer patients and the enhanced metastatic ability of cancer cells. We also showed that its inactivation by G-4 effectively reduced the up-regulation of MDR, MCL1, Survivin in YAP-overexpressing cells. Thus, we proposed a novel mechanism in which YAP augments COX-2 expression as well as its downstream targets, Survivin, MDR, MCL1, and thereby up-regulates the effect of drug resistance in CRC cells.

Recently, with the identification of more regulatory components, the Hippo pathway seems to be far from a simple linear pathway. Its activity is clearly mediated through crosstalk with other signaling pathways. The WNT, transforming growth factor-β (TGFβ)–bone morphogenetic protein (BMP), Hedgehog (HH), Notch, insulin and mTOR pathways have all been reported to functionally interact with the Hippo pathway [29].

Although both COX-2 and YAP play important role in cell proliferation, survival and tumor maintenance, whether there is cross-talk between them remains poorly understood. In the present study, we found that YAP and COX-2 were both overexpressed in CRC cells. YAP up-regulated COX-2 protein expression at the level of transcription. Deletion of the TEAD binding site in the COX-2 promoter diminished COX-2 transcriptional induction by YAP indicating that an intact TEAD binding site was necessary for YAP’s induction of COX-2. Also, YAP up-regulated COX-2 catalyzed product, PGE_2_, and downstream targets MDR, MCL1 and Survivin. These findings clearly indicate that Hippo-YAP signaling mediates the functions of COX-2/PGE_2_/EPs pathway and YAP is a nexus of the two pathways.

Having shown that there was an interaction between Hippo-YAP and COX-2 pathway and COX-2-mediated chemoresistance was regulated by YAP signaling, was there a possibility that COX-2 regulated YAP expression vice versa? Our preliminary study showed that in COX-2-overexpressing HepG_2_ cells, COX-2 knockdown reduced the expression of YAP. In addition, by overexpressing COX-2 in COX-2-low immortal THLE-3 hepatic cells, enhanced levels of COX-2 were accompanied by up-regulation of YAP expression (data not shown). These results suggested that a feedback loop may exist between YAP and COX-2.

**Fig. 13:**
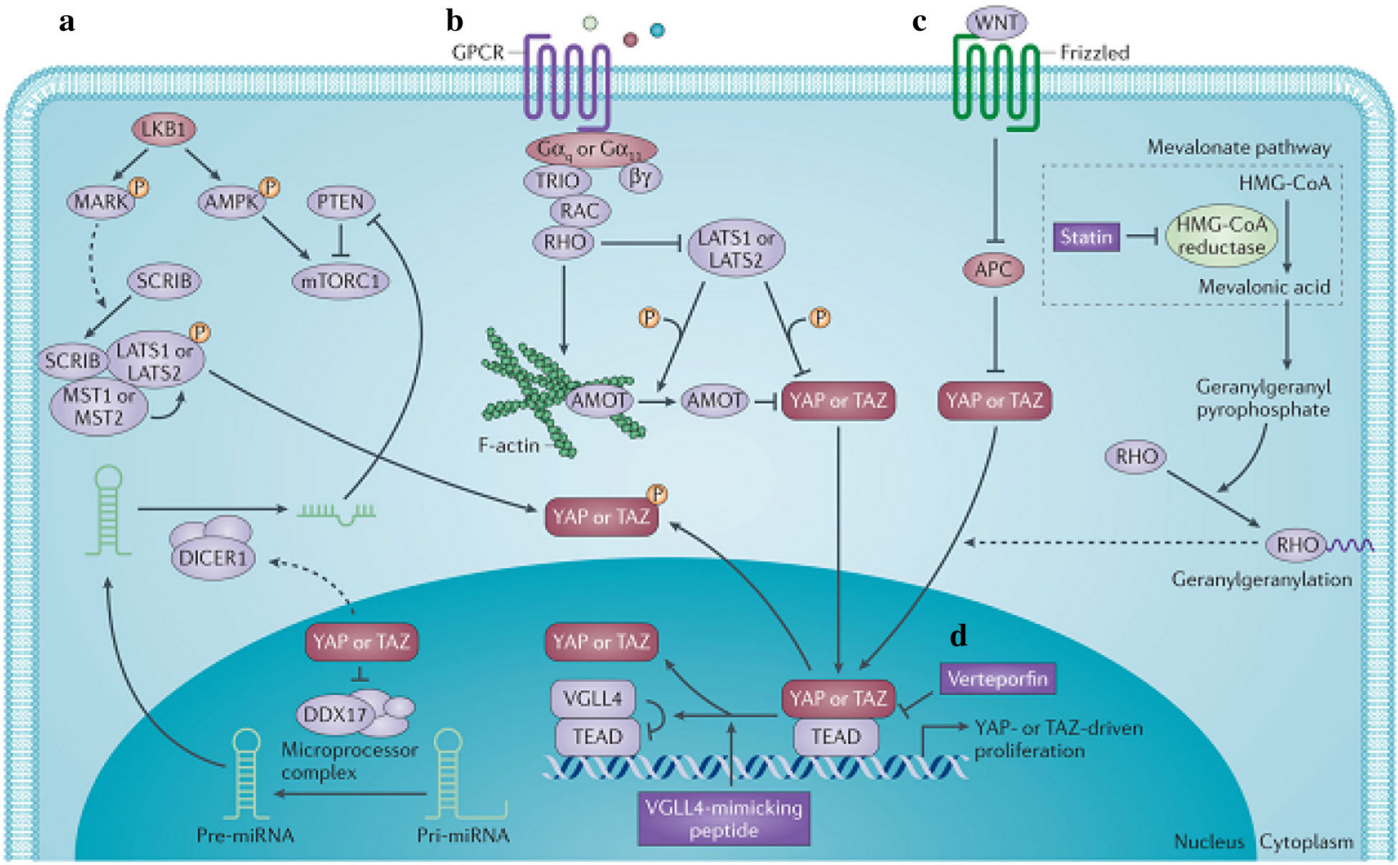
Agents that affect the Hippo pathway (Nat Rev Cancer. 2015;15(2):73-79.)

**Fig. 14:**
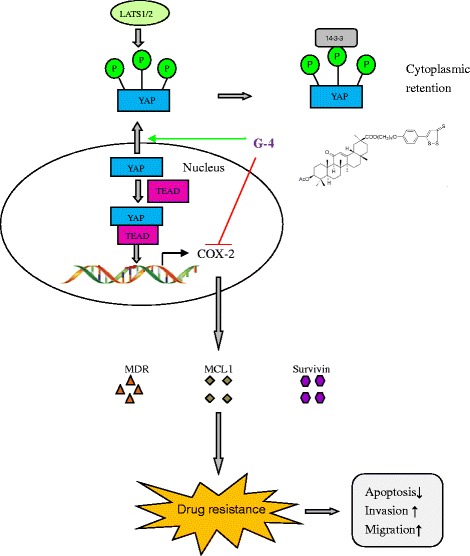
YAP mediates drug-resistance through triggering COX-2 over-expression and regulatory effects of G-4

Hydrogen sulfide-releasing non-steroidal anti-inflammatory drugs (HS-NSAIDs) are a new class of compounds with potential in alleviating gastrointestinal and cardiovascular adverse effects [30]. Some of them are now in clinical trial II. Recently, some of HS-NSAIDs have been shown with potency in inhibiting the growth of human cancers. However, studies regarding the underlying mechanism have not been abundantly carried out. In this study, we found that G-4 could drive YAP from nucleus to cytosol and promote its retention in cytosol through phosphorylation, hence affecting the downstream events such as YAP transcription. This mechanism has become one of the therapeutic targets for agents that have been found to disturb the Hippo pathway (Fig. 13). Additionally, as expected, G-4 showed direct COX-2 inhibition independent of its suppression on YAP. As a result, G-4 can be identified as a dual inhibitor of YAP and COX-2. Because YAP and COX-2 are involved in drug resistance, we further discovered that their downstream effectors such as CTGF, Cyr 61, MCL, MDR1, Survivin, Bcl-xL were down-regulated and G-4 demonstrated remarkable effect on biological behaviors of Taxol resistant cells (Fig. 14). Finally, we turned to whether YAP and COX-2 had synergistic performance in keeping resistance. Results showed that not only G-4 was more potent than VP or celecoxib (a single inhibitor of YAP or COX-2) in inducing apoptosis and reducing viability of Taxol resistant CRC cells, but also combination of shYAP and COX-2 exhibited advantages over either shYAP or shCOX-2 alone. These results point to the idea that targeting YAP and COX-2 would be more efficacious than single inhibition in overcoming drug resistance regarding YAP/COX-2 high expression and G-4 could be a novel drug candidate for successful drug resistant CRC treatment.

## Conclusions

In conclusion, this study demonstrates that YAP is an upstream regulator of COX-2 and targeting YAP-COX-2 may be a potential promising strategy to treat drug-resistant colorectal cancers. G-4 may provide a promising alternative therapeutic approach for cancer patients who are not sensitive to YAP or COX-2 inhibitor. Dual inhibitors of YAP and COX-2 may be of particular value for chemotherapeutic drug resistance in tumors with high levels of YAP/COX-2 expression.

## Additional files

Additional file 1: Figure S1. G-4 inactivates YAP in colorectal cancer cells. **a** G-4 treatment (6 h, 5, 10, 20 μM) induces YAP phosphorylation in cytosol and decreases YAP levels in nucleus of HCT8/Tax cells. **b** G-4 (10 μM) decreases YAP nuclear localization in HCT8/Tax cells. YAP subcellular localization was determined by immunofluorescence staining for endogenous YAP (green) along with DAPI for DNA (blue). ** *P* < 0.01 compared with control. (PDF 140 kb)

Additional file 2: Figure S2. G-4 disturbed YAP-TEAD interaction. **a** G-4 treatment disturbed the YAP-TEAD1 interaction in the nucleus of HCT8/Tax cells. The YAP-TEAD1 interaction was probed in cells 4 h after G-4 treatment and in untreated cells using co-IP. **b** ChIP analysis of YAP interaction with the Cyr 61 and COX-2 promoter in HCT8/Tax cells. YAP was examined in cells 4 h after G-4 treatment and in untreated cells. ** *P* < 0.01 compared with Vehicle group. (PDF 72 kb)

Additional file 3: Figure S3. G-4 (10 μM) decreased viability and suppressed cell colony formation, migration and invasion. **a** MTT assay for cell viability. HCT8 and 15/Tax cells were treated with G-4 for 48 h. **b** Effect of G-4 on cell colony formation. HCT8/Tax cells were seeded into 6-well plates and 9 days later, the colonies were stained with crystal violet, photographed (upper panel) and counted (lower panel). Scale bar: 5 mm. **c** Effect of G-4 on cell migration in HCT8/Tax cells. Cells were seeded into 6-well plates at 70–80% confluence. Cell migration was monitored by optical inspection for 24 h using a microscope and pictures were taken at 0 and 24 h (upper panel) and quantified (lower panel). **d** Effect of G-4 on cell invasion in HCT8/Tax cells. Invasion assay was conducted utilizing transwell chambers. The invaded cells were photographed (upper panel) and quantified (lower panel). ** *P* < 0.01 compared with Taxol. Taxol was applied at 1 μM in all experiments. G-4: GCCSysm-4, Tax:Taxol. (PDF 134 kb)

Additional file 4: Figure S4. YAP and COX-2 were essential for the effect of G-4 and acted synergistically to overcome the resistance. **a** Cell viability analysis in YAP or COX-2 expressing vector-transfected cells treated with G-4 (10 μM) for 48 h. **P < 0.01 compared with G-4. **b** Apoptosis and cell viability of HCT8/Tax cells treated with concentrations of 1 μM Taxol and 10 μM Verteporfin, Celecoxib, G-4 for 48 h. **c** Apoptosis and cell viability of HCT8/Tax cells after shYAP or shCOX-2 was introduced. Tax:Taxol, VP: verteporfin, Cel: celecoxib, G-4: GCCSysm-4. (PDF 66 kb)

## Abbreviations

ABC: ATP-binding-cassette transporters
BCRP: Breast-cancer-resistance protein
ChIP: Chromatin immunoprecipitation
COX-2: Cyclooxygenase 2
CRC: Colorectal cancer
HCT8 and 15/Tax cells: Taxol-resistant HCT8 and HCT15 cells
MCL: Myelocytic leukemia
MDR1/P-gp: Multidrug resistance/P-glycoprotein
MRP1: Multidrug-resistance protein 1
TEAD: TEA domain
VP: Verteporfin
YAP: Yes-associated protein
