# Supplementary figures and images for: YAP transcriptionally regulates COX-2 expression and GCCSysm-4 (G-4), a dual YAP/COX-2 inhibitor, overcomes drug resistance in colorectal cancer
Source: J Exp Clin Cancer Res. 2017 Oct 16;36:144. doi: 10.1186/s13046-017-0612-3 (PMC5644195; doi:10.1186/s13046-017-0612-3)

Supplementary material

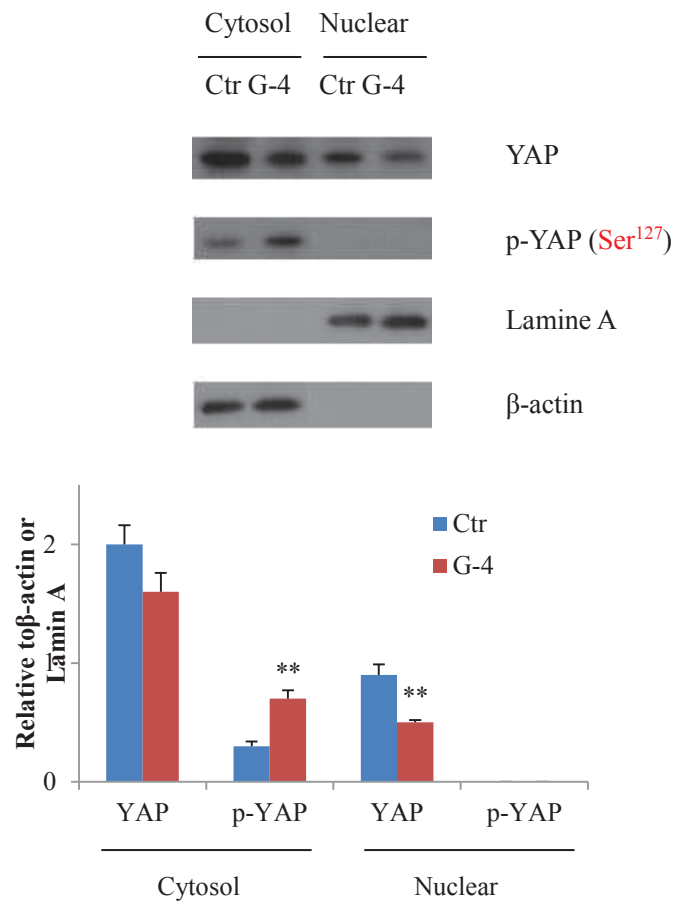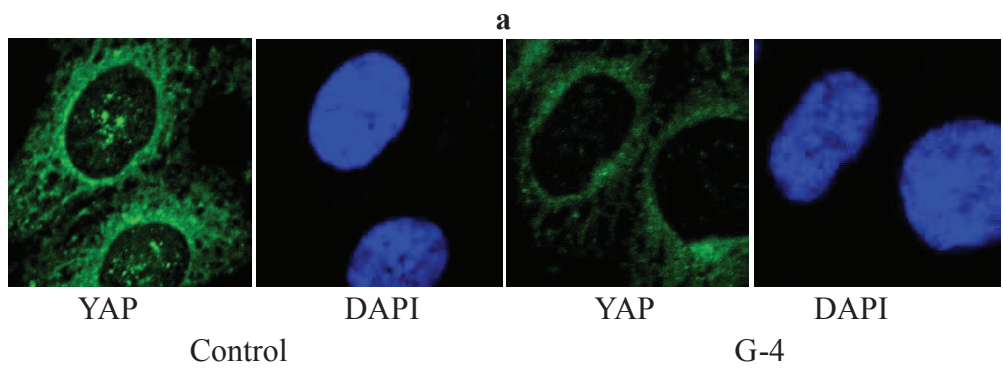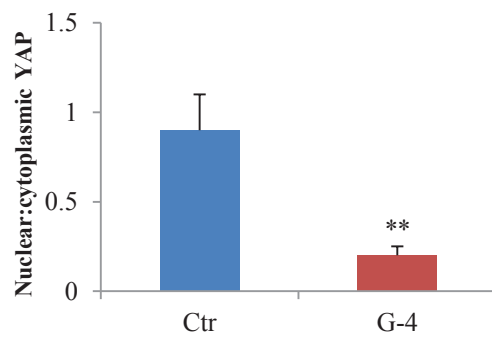

**b**

**Fig. S1**

Supplement: Supplementary file 1 — G-4 inactivates YAP in colorectal cancer cells. a G-4 treatment (6 h, 5, 10, 20 μM) induces YAP phosphorylation in cytosol and decreases YAP levels in nucleus of HCT8/Tax cells. b G-4 (10 μM) decreases YAP nuclear localization in HCT8/Tax cells. YAP subcellular localization was determined by immunofluorescence staining for endogenous YAP (green) along with DAPI for DNA (blue). ** P < 0.01 compared with control. (PDF 140 kb) [file 13046_2017_612_MOESM1_ESM.pdf]

1  
2  
3  
4  
5  
6  
7  
8  
9  
10  
11  
12  
13  
14  
15  
16  
17  
18  
19  
20  
21  
22  
23  
24  
25  
26  
27  
28  
29  
30  
31  
32  
33  
34  
35  
36  
37  
38  
39  
40  
41  
42  
43  
44  
45  
46  
47  
48  
49  
50  
51  
52  
53  
54  
55  
56  
57  
58  
59  
60  
61  
62  
63  
64  
65

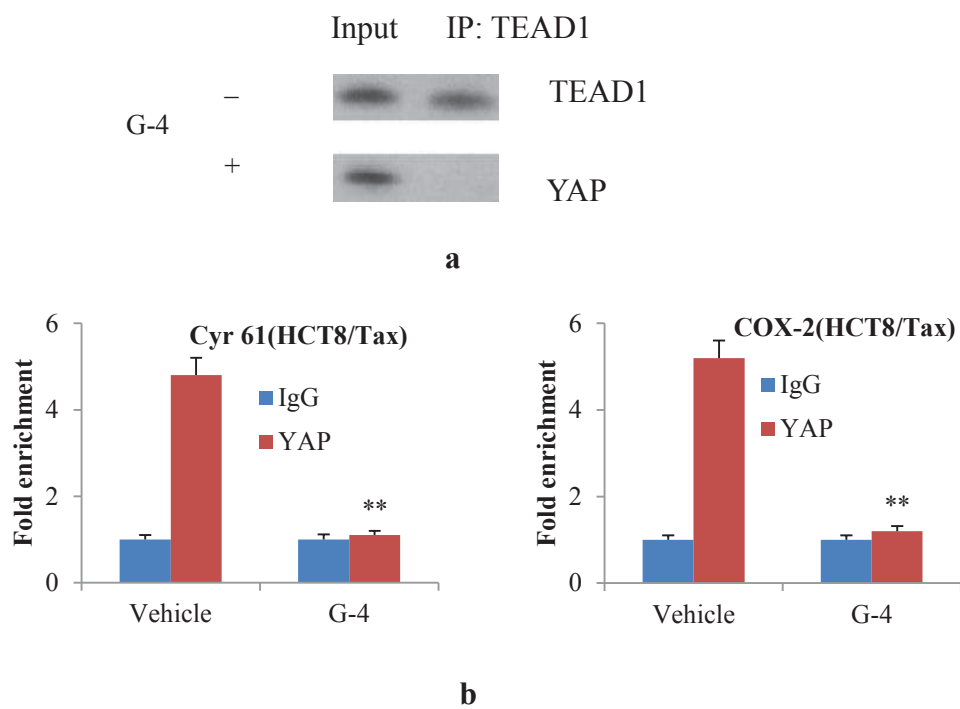

**Fig. S2**

Supplement: Supplementary file 2 — G-4 disturbed YAP-TEAD interaction. a G-4 treatment disturbed the YAP-TEAD1 interaction in the nucleus of HCT8/Tax cells. The YAP-TEAD1 interaction was probed in cells 4 h after G-4 treatment and in untreated cells using co-IP. b ChIP analysis of YAP interaction with the Cyr 61 and COX-2 promoter in HCT8/Tax cells. YAP was examined in cells 4 h after G-4 treatment and in untreated cells. ** P < 0.01 compared with Vehicle group. (PDF 72 kb) [file 13046_2017_612_MOESM2_ESM.pdf]

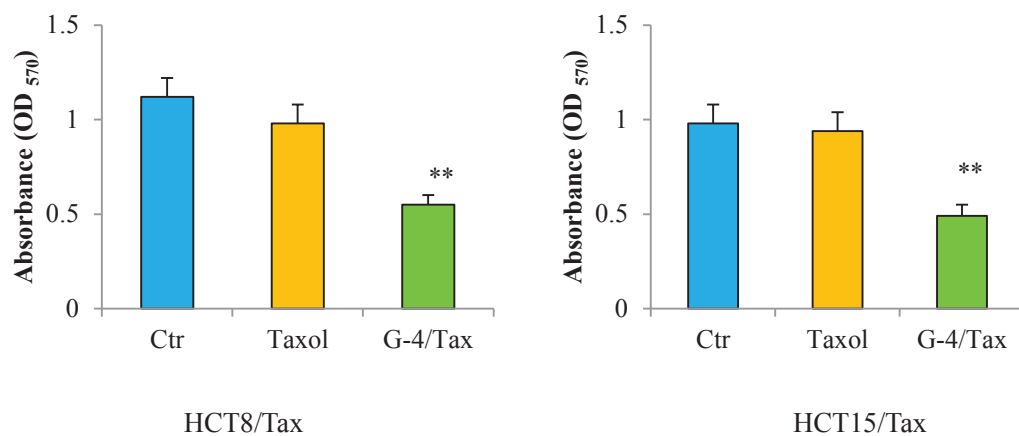

**a**

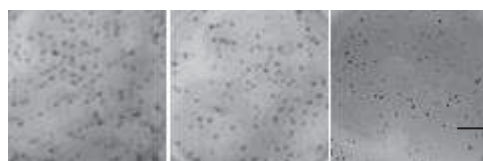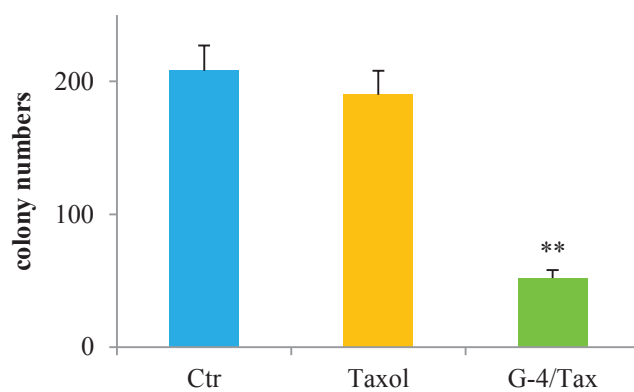

**b**

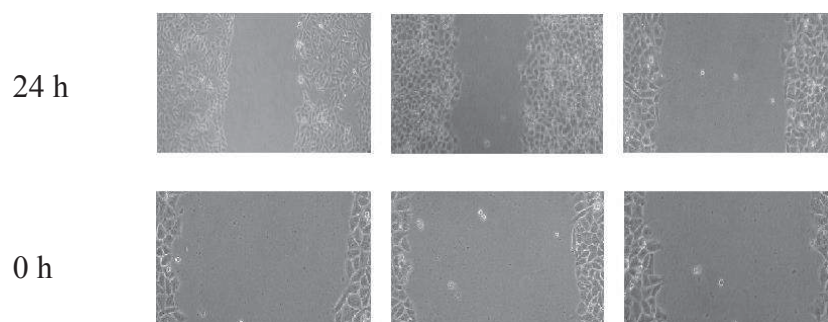

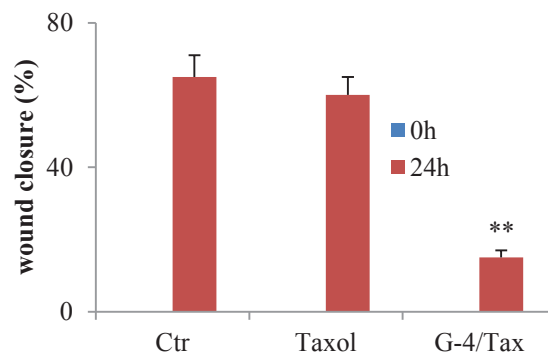

**c**

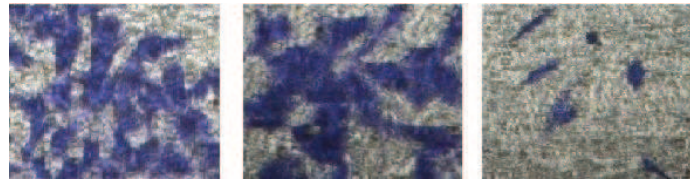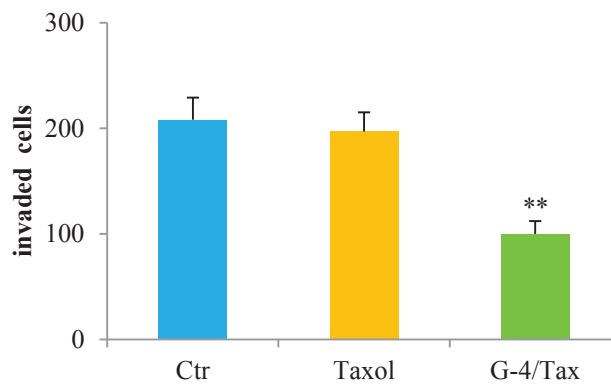

**d**

**Fig. S3**

Supplement: Supplementary file 3 — G-4 (10 μM) decreased viability and suppressed cell colony formation, migration and invasion. a MTT assay for cell viability. HCT8 and 15/Tax cells were treated with G-4 for 48 h. b Effect of G-4 on cell colony formation. HCT8/Tax cells were seeded into 6-well plates and 9 days later, the colonies were stained with crystal violet, photographed (upper panel) and counted (lower panel). Scale bar: 5 mm. c Effect of G-4 on cell migration in HCT8/Tax cells. Cells were seeded into 6-well plates at 70–80% confluence. Cell migration was monitored by optical inspection for 24 h using a microscope and pictures were taken at 0 and 24 h (upper panel) and quantified (lower panel). d Effect of G-4 on cell invasion in HCT8/Tax cells. Invasion assay was conducted utilizing transwell chambers. The invaded cells were photographed (upper panel) and quantified (lower panel). ** P < 0.01 compared with Taxol. Taxol was applied at 1 μM in all experiments. G-4: GCCSysm-4, Tax:Taxol. (PDF 134 kb) [file 13046_2017_612_MOESM3_ESM.pdf]

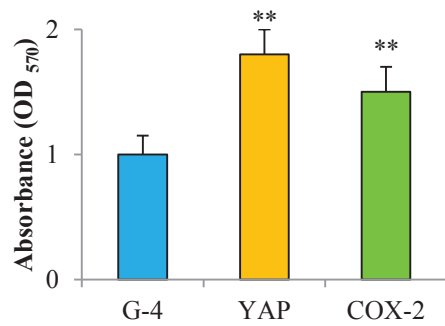

HCT8/Tax

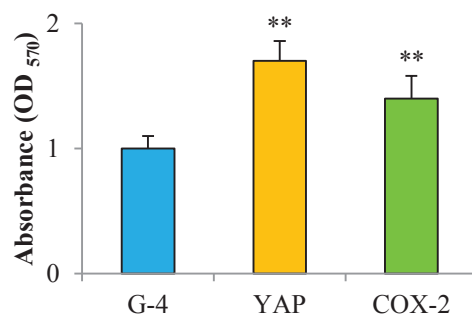

HCT15/Tax

a

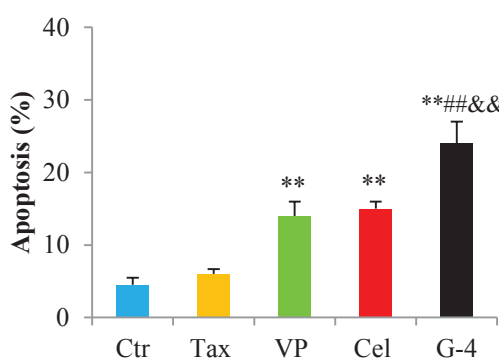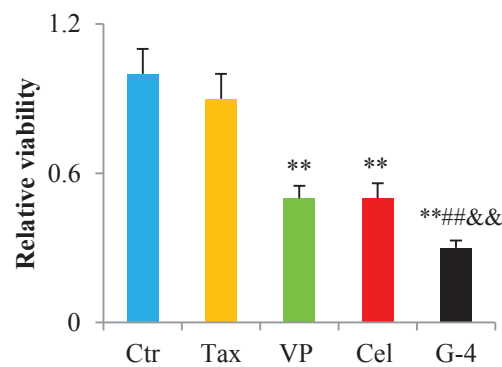

b

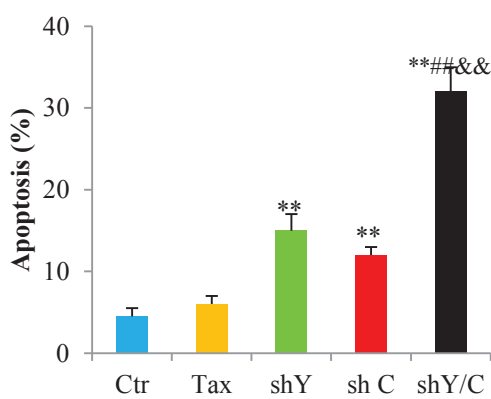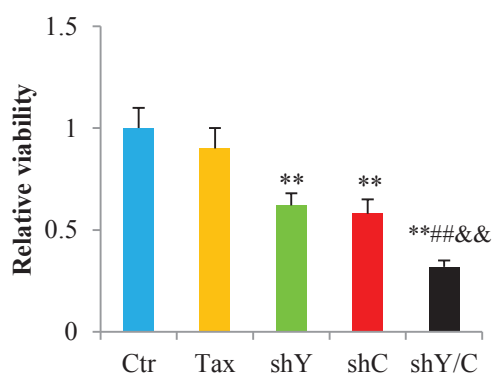

c

Fig. S4

Supplement: Supplementary file 4 — YAP and COX-2 were essential for the effect of G-4 and acted synergistically to overcome the resistance. a Cell viability analysis in YAP or COX-2 expressing vector-transfected cells treated with G-4 (10 μM) for 48 h. **P < 0.01 compared with G-4. b Apoptosis and cell viability of HCT8/Tax cells treated with concentrations of 1 μM Taxol and 10 μM Verteporfin, Celecoxib, G-4 for 48 h. c Apoptosis and cell viability of HCT8/Tax cells after shYAP or shCOX-2 was introduced. Tax:Taxol, VP: verteporfin, Cel: celecoxib, G-4: GCCSysm-4. (PDF 66 kb) [file 13046_2017_612_MOESM4_ESM.pdf]
